# Psychological vulnerability and opioid medication use disorder risk in chronic pain: the mediating role of affective states and gender differences

**DOI:** 10.3389/fpubh.2026.1810909

**Published:** 2026-09-18

**Authors:** Jesús D. Lorente, Francisco Molins, Félix Padilla, Diana Serrano-Herrera, Vicente Monsalve Dolz, Ana Mínguez, Jose De Andres, Miguel Ángel Serrano, Lucía Hipólito

**Affiliations:** 1 University Institute of Biotechnology and Biomedicine (BIOTECMED), University of Valencia, Burjassot, Spain; 2 Department of Pharmacy and Pharmaceutical Technology and Parasitology, University of Valencia, Burjassot, Spain; 3 Department of Psychobiology, University of Valencia, Valencia, Spain; 4 Department of Anaesthesia, Critical Care and Pain Medicine, Consorcio Hospital General Universitario de Valencia, Valencia, Spain; 5 Department of Surgery, University of Valencia, Valencia, Spain

**Keywords:** anhedonia, anxiety, chronic pain, depression, opioids

## Abstract

**Introduction:**

Chronic pain is a leading cause of disability and a major public health concern, frequently accompanied by anxiety and depressive symptoms that may increase vulnerability to opioid medication misuse (OMUD). While affective mechanisms linking pain intensity to the risk of suffering an opioid use disorder have been documented primarily in Anglo-Saxon populations, evidence from Southern European healthcare contexts remains limited. Replicating these pathways across healthcare systems is essential to inform prevention-oriented pain management strategies. This study examined whether negative affective states mediate the relationship between subjective pain intensity and opioid medication use disorder risk in a Spanish clinical sample and whether these associations differ by gender.

**Methods:**

Ninety-seven patients attending a specialized pain unit in Spain completed validated measures of pain intensity (visual analog scale, VAS), anxiety (Beck Anxiety Inventory, BAI), depression (Beck Depression Inventory II fast screen, BDI-FS), anhedonia (Snaith–Hamilton Pleasure Scale, SHAPS), and OMUD risk (current opioid misuse measure, COMM). Correlational, mediation (Sobel test), gender-stratified, and cluster analyses were conducted.

**Results:**

Pain intensity was positively associated with anxiety, depression, and OMUD risk. Anxiety and depression significantly mediated the association between pain and OMUD risk, with effects observed in women but not in men. The cluster analysis identified a subgroup characterized by elevated negative affect that showed significantly higher pain intensity and markedly greater OMUD risk.

**Discussion:**

These findings replicate affective mediation models in a Spanish healthcare context and underscore the systemic relevance of psychological vulnerability in chronic pain. From a public health perspective, integrating routine psychological screening in pain services may strengthen risk stratification, support safer opioid prescribing, and promote more integrated models of care. Consideration of gender differences may further enhance equity and precision in chronic pain management policies.

## Introduction

Psychological comorbidities frequently accompany chronic pain (1–4) probably because chronic pain affects the brain’s systems involved in emotion and reward (5–8). These neuroadaptations foster negative affective states that promote maladaptive coping behaviors, including opioid medication use disorder (OMUD) (4, 9–11).

Given the pressing opioid crisis and the widespread burden of chronic pain, it is crucial to better understand the contribution of emotional distress to OMUD in this population with the objective of improving quality of life and ensuring the rational use of pain medications while minimizing risks. Most of the published data supporting this hypothesis have been obtained predominantly in the US, where the opioid crisis has evolved during the last decades from the increase in the number of opioid prescriptions. Although opioid-related mortality remains lower in Europe than in the United States, contributing to the perception that Europe is safe from prescription opioid-related harms, opioid prescribing has steadily increased in several European countries, including in Spain. A closer examination reveals a progressive increase in opioid defined daily doses (DDDs) in some European countries, including Spain (12, 13). These doses do not always meet the criteria for opioid prescription (restriction of opioids for acute and cancer pain), thus increasing the risk of OMUD in the chronic pain population.

These trends in opioid prescribing underscore the need to investigate European populations because their unique healthcare system characteristics warrant a more accurate understanding of the relationship between chronic pain, emotional states, and drug use disorders. Consequently, our first objective is to investigate the co-occurrence of chronic pain and comorbid negative affective states. The second is to perform a mediation analysis examining the roles of anxiety, depression, and anhedonia in the relationship between self-reported pain and OMUD. The third is to use the cluster analysis to determine whether a specific subgroup of patients exhibits higher levels of self-reported pain and/or an increased risk of OMUD. All of these studies have been performed, for the first time, in a Spanish population cohort of patients admitted to a pain care unit. Additionally, given that gender differences have emerged as a critical factor in pain perception, emotional response, and addiction, we examine potential gender-specific differences (14–20). By performing this analysis, we aimed to provide detailed individual (“micro-”) clinical data from a Spanish cohort and analyze possible gender-dependent effects. Finally, since negative affect correlates with increased self-reported pain and/or risk of OMUD, we propose a clinical psychology evaluation for early identification of patients with chronic pain at risk, enabling preventive interventions that provide a safer use of opioids and improve overall patient care.

## Materials and methods

### Participants and procedure

Participants were recruited from the Pain Unit of the Consorcio Hospital General Universitario de Valencia (HGUV), Spain, during routine medical appointments. Of the 101 eligible patients who agreed to participate, four were excluded for not completing the questionnaires, resulting in a final sample of 97 participants (age: *M* = 57.77, SD = 13.56; women: 69.1%, *n* = 67). The inclusion criteria were chronic pain lasting at least 6 months, excluding patients with migraine and cancer-related pain, preserved the cognitive ability to complete the study questionnaires, and a current opioid prescription for pain treatment. This study was approved by the Clinical Research Ethics Committee of the Consorcio Hospital General Universitario de Valencia (registry number 11/2020) in accordance with the ethical standards of the Declaration of Helsinki and applicable laws. To minimize bias, investigators were blinded to participants’ clinical histories and had no role in their clinical management. In addition, all participant data were anonymized and coded at the time of collection, preventing identification of individuals during data collection, curation, analysis, and reporting. All participants read and signed the informed consent and completed socioeconomic questions (i.e., age, gender, and education level) and the following questionnaires:

- The Current Opioid Misuse Measure (COMM) (21) is a self-reporting assessment tool for patients with chronic pain who are prescribed opioids. It identifies the risk of aberrant medication-related behaviors. It comprises 17 items, rated on a scale from 0 to 4, reflecting behaviors over the past 30 days, with higher scores indicating a greater risk of OMUD. A score of 9 or higher is considered indicative of the risk of OMUD. Psychometric evaluation of the Spanish version of the COMM indicates good internal consistency (*α* = 0.80–0.86), satisfactory test–retest reliability, and adequate criterion validity, supporting its utility for identifying opioid misuse behaviors such as non-adherent medication use, unsanctioned dose escalation, emergency healthcare utilization, and interpersonal difficulties (22).
- The Beck Anxiety Inventory (BAI) (23) is a self-report inventory for measuring the severity of anxiety. The scale consists of 21 items describing common symptoms of anxiety. The respondent rates the extent to which each symptom has affected them over the past week on a 4-point scale. Total scores were calculated by summing item responses for all 21 items. In addition to the total score, participants were divided into the following categories: minimal anxiety (0–7), mild anxiety (8–15), moderate anxiety (16–25), and severe anxiety (26–63). The BAI demonstrated adequate psychometric properties; the instrument showed appropriate response category functioning, satisfactory item fit, a unidimensional factorial structure, and significant correlations with other anxiety measures, supporting its construct validity. Moreover, the scale demonstrated strong convergent validity and acceptable diagnostic accuracy for anxiety disorders, with a sensitivity of 85.0% and a specificity of 88.1%, as well as good internal consistency (*α* = 0.93) (24–26).
- The Beck Depression Inventory II Fast Screen (BDI) (27) is a 7-item self-reporting questionnaire for evaluating the presence and severity of depression. Each item is rated on a 4-point scale. Affective, cognitive, somatic, and vegetative symptoms are covered, reflecting the DSM-IV criteria for major depression. Scoring is achieved by adding the highest ratings to all seven items. In addition to the total score, participants were divided into the following categories: minimal depression (0–3), mild depression (4–8), moderate depression (9–12), and severe depression (13–21). The BDI Fast Screen showed good reliability and strong convergent validity across medical and general populations, with high internal consistency in the general population (*α* = 0.84), strong correlation with the BDI (*r* = 0.85), and excellent screening accuracy in patients with chronic pain, where a cutoff score of ≥4 corresponded to the BDI cutoff score of 19 recommended in the BDI manual for detecting depression. Its one-factor structure and measurement invariance across age and gender further support its usefulness as a brief and reliable depression screener, particularly in medically ill populations (28, 29).
- The Snaith–Hamilton Pleasure Scale (SHAPS) (30) is a self-reporting questionnaire designed to assess anhedonia. It consists of 14 items, each describing a pleasurable experience, that respondents rate on a 4-point scale, indicating how much they agree with each statement over the past few days. A score of 3 or higher is generally considered indicative of significant anhedonia. The Spanish version of the Snaith–Hamilton Pleasure Scale (SHAPS) has shown adequate reliability and validity for assessing anhedonia, with acceptable internal consistency in clinically depressed samples (Cronbach’s *α* = 0.77) and evidence of construct, convergent, concurrent, and discriminant validity (42).
- The visual analog scale (VAS) of pain (46) consisted of a 10-cm horizontal line with two anchors—“no pain sensation” and “most intense pain imaginable”. Participants indicated their pain level during the last month of the assessment by marking a spot on the line. To obtain a numerical score (0–10) for this rating, a ruler was used to measure the distance (in cm). Scores of 3–7 are considered moderate pain, while scores of 8–10 indicate severe pain. VAS has shown strong validity and reliability for pain assessment, particularly in chronic and experimental pain contexts, with excellent test–retest reliability reported for osteoarthritic knee pain (ICC = 0.97), supporting its stability and precision as a pain measurement tool (31, 41).

### Statistical analyses

Data are shown as mean ± SD. Outliers were identified using the 2.5-standard-deviation method, and normality was assessed using the Kolmogorov–Smirnoff test with Lilliefor’s correction. Exploratory analyses were conducted to examine the distribution of variables and their interrelations with gender. Differences between men and women in demographic, clinical, and psychological variables were assessed using independent-sample *t*-tests. Mediation analyses were performed to investigate whether anxiety, depression, and anhedonia mediate the association between subjective pain and opioid consumption. The Sobel test was utilized to check the statistical significance of the mediation models. Finally, clustering analyses were conducted to examine how participants were grouped based on their levels of anxiety, depression, and anhedonia. These clusters were then analyzed to identify differences in pain and COMM and to explore relationships between these variables within clusters using the multivariate analysis of variance (MANOVA).

The significance level (*α*) was set at 0.05, and the partial eta squared (*η*^2^*_p_*) indicated the effect size. All analyses were conducted using IBM SPSS Statistics 25.

## Results

### Descriptive and comparative analyses of main variables

Table 1 shows descriptive and comparative analyses of the main variables. Our sample possessed a moderate education level (3.35 ± 1.26 out of 6) and a mean age of 57.77 ± 13.55. Referring to pain-related variables, participants reported an average pain duration of 10.79 ± 10.71 years. Moreover, over the past month, they experienced pain at a level corresponding to moderate-to-severe intensity (7.79 ± 1.69 out of 10), as measured by the VAS. Assessment of opioid use with the COMM revealed a mean score (13.16 ± 10.71) above the clinical cutoff (>9), indicating problematic opioid use within the sample (21). Regarding emotional variables, anxiety levels were moderate (18.79 ± 13.69), while depression levels were mild (5.47 ± 4.79), and anhedonia did not exceed the cutoff score of 3, which would indicate a problematic level (1.33 ± 2.10). Furthermore, when we compared using an independent *t*-test, almost all the variables did not show significant differences; however, we found that anxiety levels (BAI) and pain perception (VAS) were significantly higher in women than in men (*p* = 0.006 and *p* = 0.032, respectively) (Table 1). Therefore, these preliminary differences between men and women point out the importance of including gender analyses in this study.

**Table 1:** Descriptive statistics.

Variables	Total (*n* = 97)	Men (*n* = 32)	Women (*n* = 65)
Age	57.77 ± 13.55	54.80 ± 12.94	59.10 ± 13.71
Educational level	3.35 ± 1.26	3.50 ± 1.20	3.28 ± 1.30
Chronic pain duration (years)	10.79 ± 10.88	8.67 ± 8.94	11.75 ± 11.58
COMM	13.16 ± 10.71	11.90 ± 9.71	13.73 ± 11.17
BAI	18.79 ± 13.69	13.10 ± 9.58	21.34 ± 14.48
BDI	5.47 ± 4.78	4.43 ± 3.79	5.94 ± 5.13
SHAPS	1.33 ± 2.10	0.83 ± 1.42	1.55 ± 2.32
Last-month VAS of pain	7.79 ± 1.69	7.25 ± 1.84	8.05 ± 1.58

Mean ± SD; Mean educational level ranges from 1 (without studies) to 6 (University studies).

Age, education level, pain duration in years, AUDIT, COMM, BAI, BDI, SHAPS, last-month, and -day VAS are represented as mean ± SD; df, degrees of freedom.

To explore relationships among variables, correlation analyses were performed for all variables in the study. Figure 1A shows that almost all variables were significantly related to each other (values of the Pearson’s correlation coefficient and *p*-values are detailed in the heat map of Figure 1A). Specifically, subjective pain, as assessed by the last-month VAS, was significantly associated with COMM scores, underscoring the association between pain intensity and problematic opioid use (Figures 1A,B). While the BAI and BDI showed low-to-moderate associations with subjective pain, they exhibited strong positive correlations with COMM scores. Additionally, anhedonia demonstrated a moderate relationship with opioid consumption, further supporting the connection between affective factors and misuse risk. Finally, a strong correlation was found between anxious and depressive symptomatology, and moderate correlations were observed between depressive or anxious symptomatology and anhedonia.

**Figure 1:**
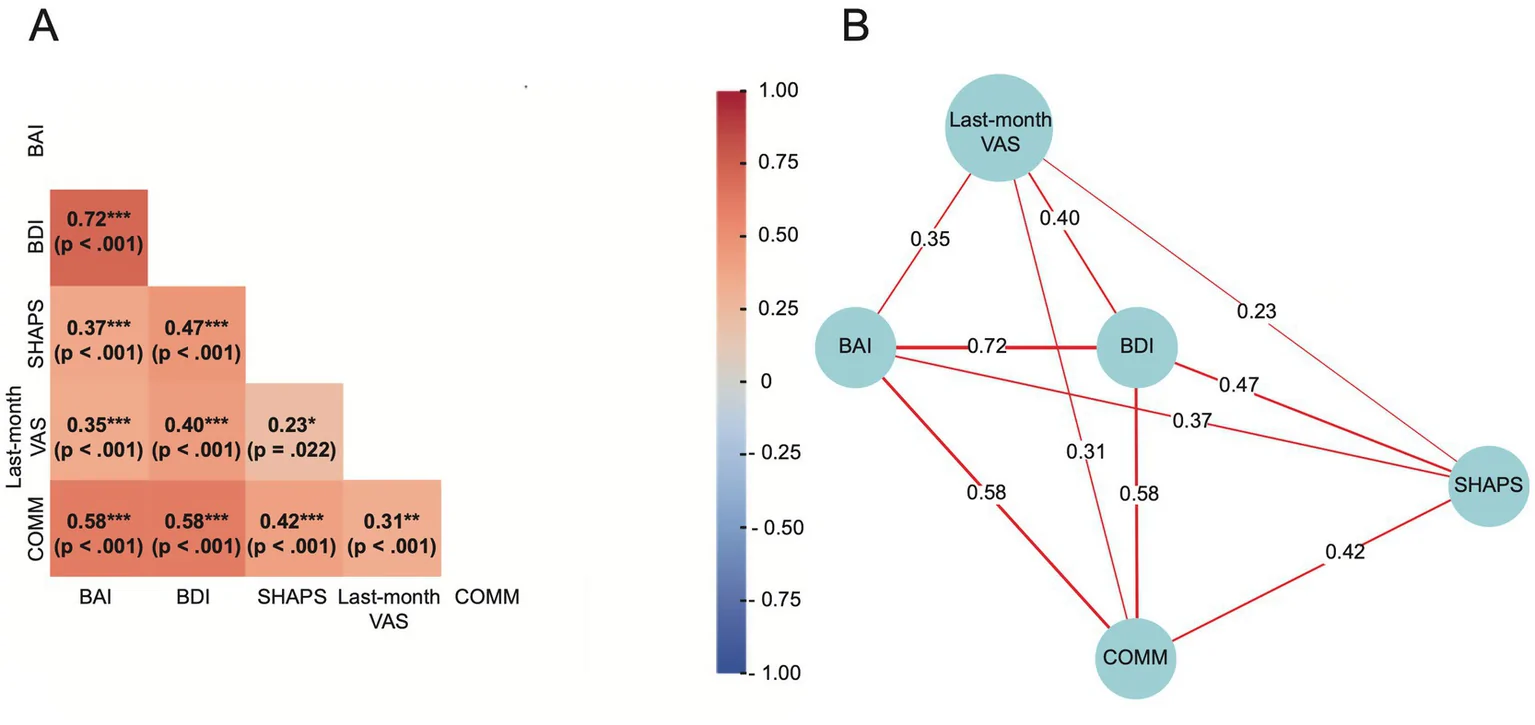
A heat map and a network of the correlations between BAI, BDI, SHAPS, VAS, and COMM. **(A)** A heat map with a color gradient indicating correlation intensity. Values in each cell represent Pearson’s correlation coefficient, and asterisks denote significant correlations (*** significant at the 0.001 level; ** significant at the 0.01 level; * significant at the 0.05 level). **(B)** A network that enhances understanding of the previous heat map by visually depicting connections among variables. The edges vary in thickness according to the strength of their correlation. Generated in Python using Matplotlib.

These correlations were further examined separately for each gender. As shown in Figures 2A,B, women exhibited similar patterns of association among the primary variables. In contrast, in men (Figures 2C,D), several correlations did not reach statistical significance. Specifically, the associations between anxious or depressive symptomatology and subjective pain did not reach statistical significance, although they demonstrated a trend toward significance (*p*-values ranging from 0.074 to 0.087). Consistent with this pattern, no significant correlations were detected between subjective pain and opioid consumption. This pattern suggests that, while pain intensity in women is embedded within a broader affective-opioid risk pathway, in men, affective symptoms and OMUD risk may operate more independently of subjective pain levels.

**Figure 2:**
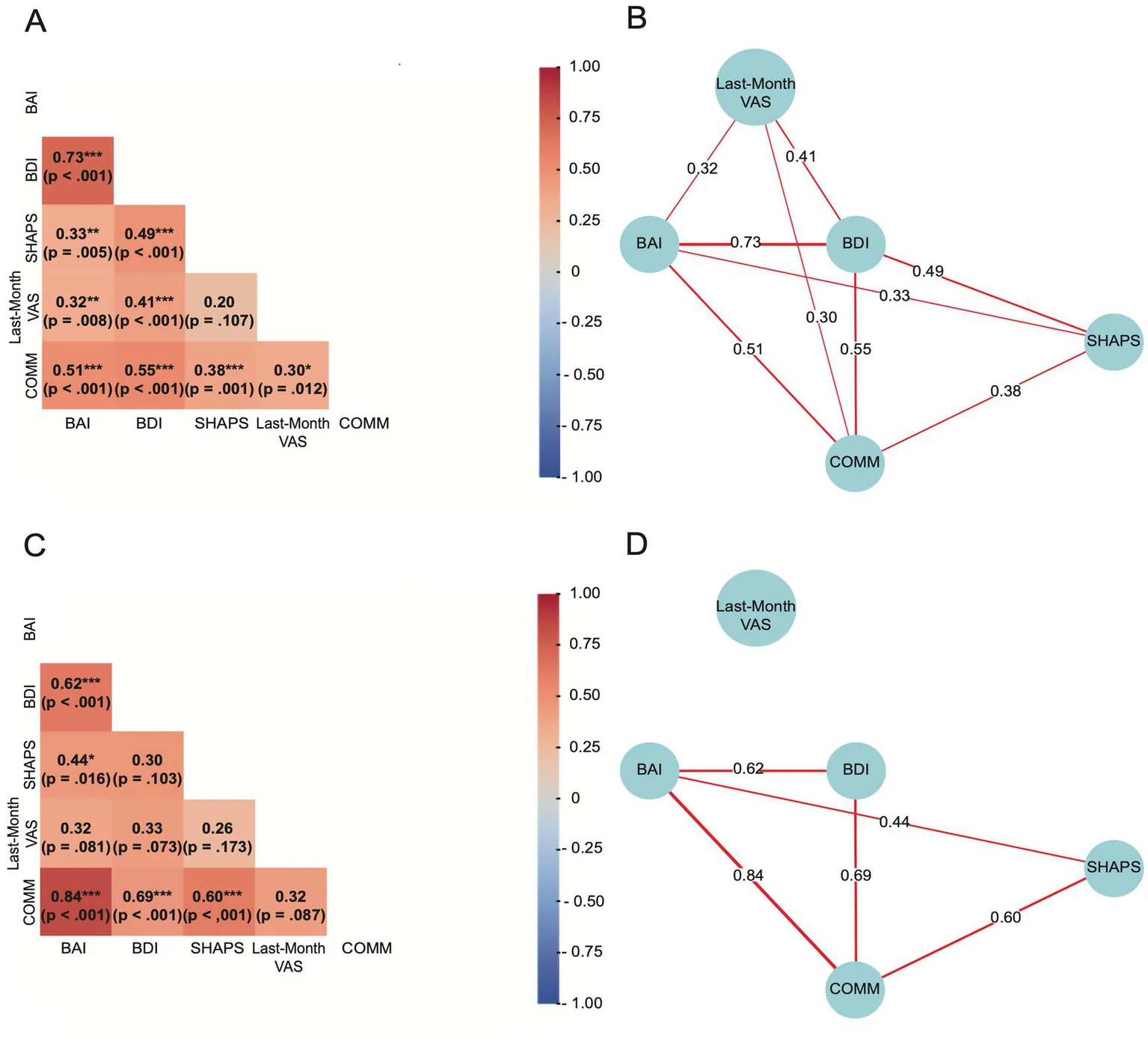
A heat map and a network of the correlations between BAI, BDI, SHAPS, VAS, and COMM, by sex. Heat maps for women **(A)** and men **(B)** with a color gradient indicating correlation intensity. Values in each cell represent Pearson’s correlation coefficient, and asterisks denote significant correlations (*** Significant at the 0.001 level; ** significant at the 0.01 level; * significant at the 0.05 level). Network that enhances the comprehension of the previous heat map by visually depicting the connections between variables. **(C)** For women; **(D)** For men. The edges vary in thickness according to the strength of their correlation. Generated in Python using Matplotlib.

### Do anxiety, depression, and anhedonia mediate the relationship between subjective pain and risk of opioid medication use disorder?

Based on the observed correlations between emotional variables and pain, we proceeded to examine the hypothesis that pain contributes to opioid consumption via these affective states in patients with chronic pain. To this end, mediation analyses were performed to determine whether anxiety, depression, and anhedonia served as mediators of the relationship between pain, assessed over the past month, and scores on the COMM, in accordance with established criteria for mediation. The significance of the mediation effect was evaluated using the Sobel test.

#### Anxiety symptoms mediate subjective pain and opioid consumption

First, subjective pain was significantly associated with the COMM score, *B* = 0.06, SE = 0.23, *t*(95) = 2.46, *p* = 0.015. Second, subjective pain was also associated with anxiety symptoms, *B* = 2.78, SE = 0.78, *t*(95) = 3.55, *p* = 0.001. Third, these symptoms were significantly associated with the COMM score, *B* = 0.46, SE = 0.06, *t*(95) = 7.11, *p* < 0.001. Finally, the relationship between subjective pain and COMM score disappeared (*B* = 0.70, SE = 0.56, *t*(94) = 1.24, *p* = 0.218) when controlling for anxiety *B* = 0.44, SE = 0.07, *t*(94) = 6.28, *p* < 0.001. Furthermore, the Sobel test indicated that this mediation was significant, *z* = 3.1, *p* = 0.001.

This mediation model was replicated separately for women and men. In women, similar results were obtained, indicating that anxiety mediated the relationship between pain and COMM scores (Sobel test: *z* = 2.3, *p* = 0.021). Thus, subjective pain was significantly associated with opioid consumption, *B* = 1.99, SE = 0.86, *t*(65) = 2.31, *p* = 0.024. Subjective pain was also associated with anxiety symptoms, *B* = 2.79, SE = 1.08, *t*(65) = 2.57, *p* = 0.012. These symptoms were significantly associated with COMM scores, *B* = 0.41, SE = 0.08, *t*(65) = 5.04, *p* < 0.001. Finally, the association between subjective pain and COMM scores was no longer statistically significant (*B* = 0.91, SE = 0.79, *t*(64) = 1.15, *p* = 0.252), when controlling for anxiety, *B* = 0.38, SE = 0.09, *t*(64) = 4.47, *p* < 0.001. Regarding the samples collected from men, a mediation analysis was not performed because the criteria for this analysis (i.e., a simple correlation between the variables) were not met.

#### Depression symptoms mediate subjective pain and opioid consumption

As indicated in the previous analysis, subjective pain over the previous month was linearly related to COMM scores. Second, subjective pain was also associated with depression symptoms, *B* = 1.09, SE = 0.27, *t*(95) = 3.94, *p* < 0.001. Third, these symptoms were significantly associated with COMM scores, *B* = 1.34, SE = 0.18, *t*(95) = 7.40, *p* < 0.001. Finally, the relationship between subjective pain and COMM scores was not significant, *B* = 0.54, SE = 0.57, *t*(94) = 0.94, *p* = 0.347, when controlling for depression, *B* = 1.26, SE = 0.19, *t*(94) = 6.51, *p* < 0.001. Furthermore, the Sobel test indicated that this mediation was also significant, *z* = 3.44, *p* < 0.001.

As indicated in the previous section, this mediation model was replicated separately for women and men. In women, very similar results were obtained, indicating that depression mediated the relationship between perceived pain and COMM scores (Sobel test: *z* = 2.8, *p* = 0.004). Thus, as previously reported, subjective pain was significantly associated with the COMM score. Subjective pain was also associated with depression symptoms, *B* = 1.25, SE = 0.38, *t*(65) = 3.23, *p* = 0.002. These symptoms were significantly associated with COMM scores, *B* = 1.24, SE = 0.21, *t*(65) = 5.73, *p* < 0.001. Finally, the relationship between subjective pain and the COMM score disappeared, *B* = 0.51, SE = 0.79, *t*(64) = 0.64, *p* = 0.52, when controlling for depression, *B* = 1.19, SE = 0.23, *t*(64) = 5.06, *p* < 0.001. In line with anxiety, the mediation analysis for men was not performed because the criteria for this analysis (i.e., simple correlation between the variables) were not met.

#### Anhedonia symptoms mediate between subjective pain and opioid consumption

As indicated in the previous analysis, subjective pain was linearly associated with COMM scores. Second, subjective pain was also associated with anhedonia symptoms, *B* = 0.287, SE = 0.12, *t*(95) = 2.31, *p* = 0.023. Third, these symptoms were significantly associated with the COMM score, *B* = 2.15, SE = 0.47, *t*(95) = 4.54, *p* < 0.001. Finally, the relationship between subjective pain and the COMM score was debilitated, *B* = 1.44, SE = 0.59, *t*(94) = 0.94, *p* = 0.016, when controlling for anhedonia, *B* = 1.88, SE = 0.47, *t*(94) = 3.96, *p* < 0.001. Furthermore, the Sobel test indicated that this mediation was also significant, *z* = 2.11, *p* < 0.017. In this case, it was not possible to replicate the mediation analyses by separating the data by gender because the necessary criteria for mediation was not met. Specifically, for both women and men, there was no significant linear relationship between subjective pain (independent variable) and anhedonia (proposed mediator), which prevents further examination of the other relationships.

### Cluster analysis for affective states and their differences in pain and opioid consumption

Given that emotional variables are related to perceived pain (at least in women) and to COMM scores, we next applied a cluster analysis to classify patients based on the combination of these variables. The objective is to identify clinically meaningful subgroups of patients with chronic pain for tailored intervention. The K-means method, based on the similarity or distance of patients’ scores in anxiety, depression, and anhedonia, was used to extract clusters. The silhouette score revealed that the optimal solution was between two clusters (silhouette score = 0.504). Specifically, as shown in Figure 3, we identified one group with impaired affective states (high anxiety, depression, and anhedonia; negative affect) and another with moderate or mild levels (neutral affect).

**Figure 3:**
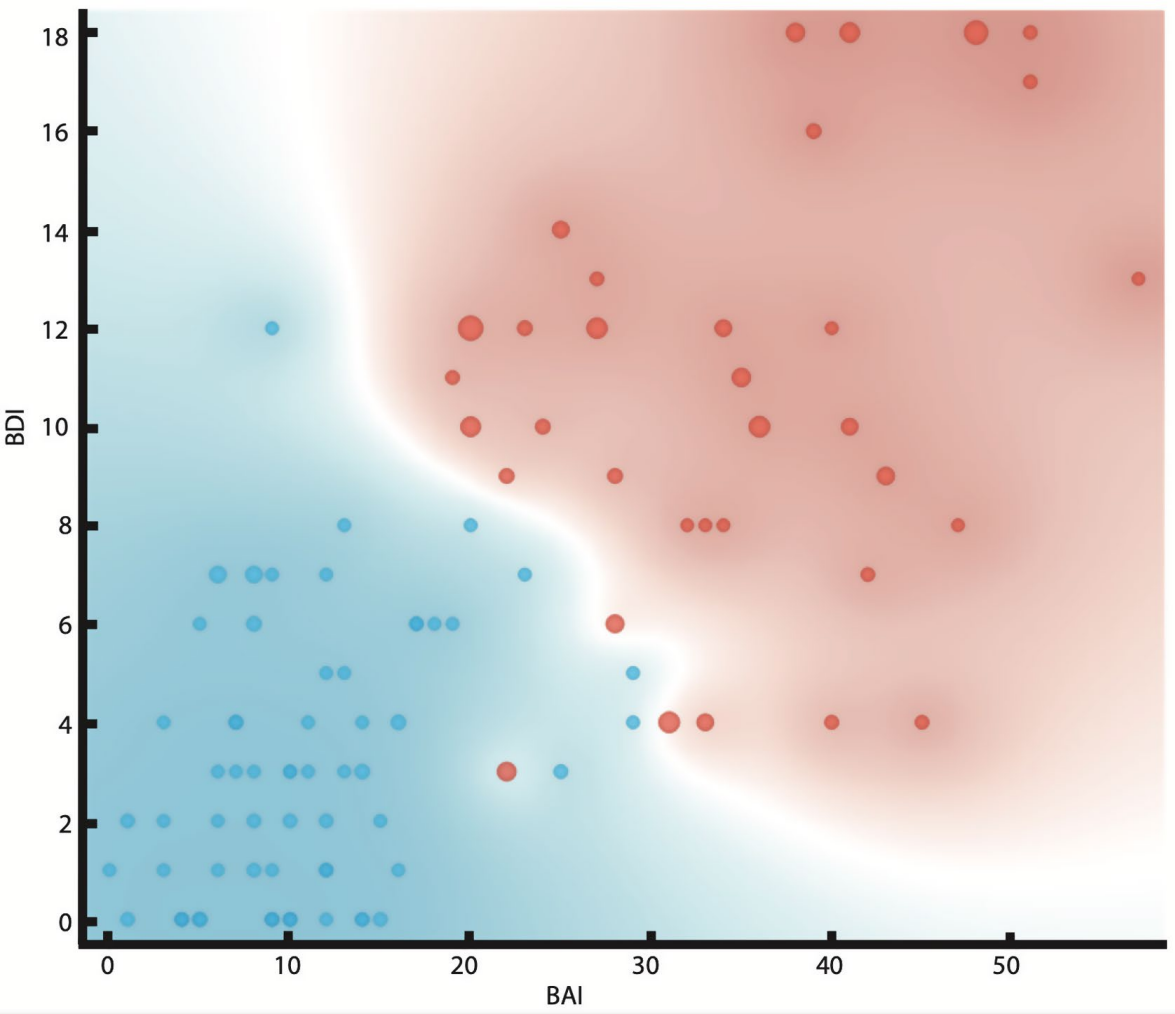
Affective state clusters: anxiety scores (BAI) on the *x*-axis and depression scores (BDI) on the *y*-axis. The size of the points represents the level of anhedonia (SHAPS): the larger the size, the higher the anhedonia. The 2-cluster solution: in red, the highest levels of anxiety, depression, and anhedonia; in blue, the lowest levels. Adaptation from the original obtained from SPSS IBM 25.

Subsequently, a MANOVA was conducted to compare the subjective pain levels and COMM scores. As shown in Table 2, the cluster characterized by negative affectivity reported significantly higher levels of subjective pain and, notably, elevated COMM scores. The effect size for opioid OMUD was moderate, underscoring the clinical relevance of this finding and indicating an increased risk of problematic opioid use in this subgroup.

**Table 2:** Differences in subjective pain (last-month and -day VAS) and opioid misuse measurement (COMM) by affective states (2-cluster solution), sex, and their interaction.

2 clusters-solution	Neutral affect (64)	Negative affect (33)			*F*	Df intra	Df error	*p-*value	*η* ^2^ *_p_*
Last-month VAS	7.38 ± 1.85^a^	8.60 ± .93^a^			6.15	1	93	0.015*	689
COMM	8.56 ± 6.11^a^	22.09 ± 12.11^a^			49.33	1	93	<0.001***	1

	Men (30)	Women (67)							
Last-month VAS	7.25 ± 1.84	8.05 ± 1.58			0.678	1	93	0.413	0.129
COMM	11.90 ± 7.71^a^	13.73 ± 11.16^a^			5.077	1	93	0.027*	0.606

	Men neurtral affect (26)	Women neutral affect (38)	Men negative affect (4)	Women negative affect (29)					
Last-month VAS	7.08 ± 1.91	7.59 ± 1.80	8.34 ± 0.48	8.64 ± 1.58	0.071	1	93	0.79	0.058
COMM	8.88 ± 5.53^a^	8.34 ± 6.54^b^	31.50 ± 8.06 ^ac^	20.79 ± 12.09^bc^	4.145	1	93	0.045	0.522

Mean ± SD by affective state clusters (*n*); df, degrees of freedom; * Significance at the 0.05 level; ** Significance at the 0.01 level; *** Significance at the 0.001 level. When comparing the interaction of variables, if the same letter appears next to both the mean and the SD values, this indicates that there were significant differences between the two groups.

We have also performed MANOVA analyses, dividing the obtained clusters by gender (Table 2). MANOVA analyses in this case found similar COMM score results for men and women when compared with the results obtained, including all participants. In fact, both men and women with negative affect scored significantly higher in COMM than their respective neutral effect groups. Although perceived pain levels were higher in participants with negative affect, these differences did not reach statistical significance in either men or women, possibly due to the limited sample size in some groups.

## Discussion

The present study provided the first evidence in a Spanish clinical sample that negative affective states are associated with both subjective pain intensity and OMUD risk in people with chronic pain. Anxiety and depressive symptoms mediated the association between pain intensity and OMUD in women, while cluster analyses identified a subgroup characterized by elevated negative affect that showed significantly greater OMUD risk. Although the predictive value of these findings requires confirmation in future studies, they suggest that affective vulnerability may represent a clinically relevant pathway linking chronic pain and opioid misuse risk, supporting the potential value of psychological assessment and intervention as part of preventive pain management strategies.

The present research reinforces a clear association between anxiety-depressive symptomatology and subjective pain levels in accordance with previous research (32–34) and underscores the importance of incorporating these variables into the diagnosis and treatment of chronic pain, particularly from a biopsychosocial perspective. In our study, anhedonia, one of the most extensively studied symptoms in mood disorders, was also associated with perceived pain levels, a finding that was consistent with previous reports (35, 36, 44). Furthermore, in agreement with previous findings from United States populations, OMUD risk was positively associated with anxiety-depressive symptomatology in our chronic pain cohort. Importantly, the mean COMM scores in our sample exceeded the established cutoff score of 9, indicating a clinically relevant level of opioid misuse risk among participants. These findings are particularly relevant in European healthcare systems, where opioid-related harms remain comparatively lower than in the United States, yet opioid prescribing continues to increase, including in Spain (12, 13). In this context, individual-level clinical data are essential to better understand early vulnerability pathways associated with opioid misuse risk before crisis-level patterns emerge.

In this study, we also explored whether anxiety-depressive symptomatology mediates the relationship between subjective pain levels and OMUD risk. Although previous literature has not consistently confirmed gender differences in these associations, our data suggest that affective mediation pathways may be particularly relevant for women. Gender is a critical variable to consider, as substantial evidence indicates gender differences in pain perception, analgesic properties of treatments, and prognosis of pain-related conditions (18, 19, 37, 38). However, the specific gender-related effects on the correlations between negative affective states, pain perception, and the risk of OMUD have not been extensively explored. In our sample, women exhibited a significant association between subjective perceived pain, anxiety-depressive symptomatology, and COMM scores, with anxiety and depressive symptoms mediating the relationship between pain and opioid misuse risk. In men, however, anxiety and depressive symptoms were associated with COMM scores but not with subjective pain; therefore, no mediation effect was observed. These findings emphasize the importance of considering gender as a relevant variable when monitoring opioid treatment and designing targeted interventions for chronic pain.

Although the reduced number of men may have limited the statistical power to detect gender-specific effects, our findings support a relationship between negative affective states and prescription opioid misuse risk in both men and women. Given the high prevalence of negative affective profiles among women, affective vulnerability may represent a particularly relevant pathway contributing to OMUD risk in this group. Therefore, the identification of anxiety-depressive symptomatology in patients with chronic pain, considering gender, emerges as a valuable tool for identifying modifiable affective risk factors to support recovery-oriented and safe pain management pathways, as negative affect is among the most significant risk factors for substance use and relapses in substance use disorders (2, 39, 43). Consistent with this interpretation, cluster analysis further confirmed that patients with negative affective profiles showed significantly higher OMUD risk, extending the role of emotional distress not only in pain experience but also in vulnerability to problematic opioid medication use. Although some gender-specific differences emerged, the limited number of men prevented categorical conclusions. Nevertheless, these findings highlight the importance of targeted psychological assessment and intervention in patients with chronic pain with anxiety-depressive symptomatology and anhedonia in order to detect and manage affective states associated with both pain and OMUD risk. In this regard, integrating psychological techniques into standard chronic pain treatment protocols has been associated with an improved quality of life, greater pain relief, reduced risk of disability, and lower levels of fear-avoidance behaviors (40).

Beyond these clinical benefits, our findings support the integration of routine psychological screening into chronic pain services to identify patients at an increased risk of OMUD. A brief assessment of anxiety, depression, and anhedonia may help guide safer opioid prescribing and promote more preventive, interdisciplinary approaches to chronic pain care, particularly in healthcare systems where early detection of opioid-related risk remains essential to avoid future escalation.

This research has some limitations. First, although we have not conducted a *post-hoc* power analysis, the lower number of men than women is a limitation, as it may affect the interpretation of gender differences. However, this imbalance reflects the usual distribution of chronic pain, which is more prevalent in women than in men in Spain and other developed countries. Second, the inclusion of different pain conditions, particularly neuropathic pain, should also be considered a limitation, as these conditions may be associated with distinct psychological profiles of anxiety-depressive symptomatology. Finally, because this is a cross-sectional study, causal relationships between variables cannot be established. Nevertheless, the associations observed here may inform future longitudinal studies aimed at clarifying the directionality and potential causal associations between pain, negative affective states, and OMUD risk.

In conclusion, this study provides novel evidence on the interplay between affective vulnerability, subjective pain, and OMUD risk in Spanish patients with chronic pain. Anxiety-depressive symptomatology and anhedonia are strongly associated with opioid misuse risk, supporting the role of emotional dysregulation as a relevant mechanism linking chronic pain and maladaptive opioid-related behaviors. Importantly, these findings replicate previously described associations between affective vulnerability and OMUD risk in a Southern European population and suggest that such vulnerability may be particularly relevant in women, who show a higher prevalence of negative affective profiles. Importantly, these results extend the role of emotional factors beyond their contribution to pain experience, highlighting their potential involvement in the risk of problematic opioid use during chronic pain treatment. From a clinical perspective, these findings support the incorporation of routine psychological assessments into chronic pain management, particularly when opioid pharmacotherapy is being considered. Screening for anxiety, depression, and anhedonia may help identify patients at increased risk of OMUD and guide more individualized, preventive, and interdisciplinary interventions. In this sense, psychological therapies should not be viewed as secondary or optional components of care but as central elements of safer and more comprehensive pain management strategies aimed at improving patient outcomes while reducing opioid-related risk.

## Data Availability

The raw data supporting the conclusions of this article will be made available by the authors, without undue reservation.
